# Publisher Correction: Heterogeneous SARS-CoV-2 humoral response after COVID-19 vaccination and/or infection in the general population

**DOI:** 10.1038/s41598-022-13938-z

**Published:** 2022-06-07

**Authors:** Fabrice Carrat, Paola Mariela Saba Villarroel, Nathanael Lapidus, Toscane Fourié, Hélène Blanché, Céline Dorival, Jérôme Nicol, Jean-François Deleuze, Olivier Robineau, SAPRIS-SERO Study Group, Fabrice Carrat, Marie Zins, Gianluca Severi, Mathilde Touvier, Hélène Blanché, Jean-François Deleuze, Xavier de Lamballerie, Clovis Lusivika-Nzinga, Gregory Pannetier, Nathanael Lapidus, Isabelle Goderel, Céline Dorival, Jérôme Nicol, Olivier Robineau, Sofiane Kab, Adeline Renuy, Stéphane Le-Got, Céline Ribet, Mireille Pellicer, Emmanuel Wiernik, Marcel Goldberg, Fanny Artaud, Pascale Gerbouin-Rérolle, Mélody Enguix, Camille Laplanche, Roselyn Gomes-Rima, Lyan Hoang, Emmanuelle Correia, Alpha Amadou Barry, Nadège Senina, Julien Allegre, Fabien Szabo de Edelenyi, Nathalie Druesne-Pecollo, Younes Esseddik, Serge Hercberg, Mélanie Deschasaux, Hélène Blanché, Jean-Marc Sébaoun, Jean-Christophe Beaudoin, Laetitia Gressin, Valérie Morel, Ouissam Ouili, Jean-François Deleuze, Laetitia Ninove, Stéphane Priet, Paola Mariela Saba Villarroel, Toscane Fourié, Souand Mohamed Ali, Abdenour Amroun, Morgan Seston, Nazli Ayhan, Boris Pastorino, Mathilde Touvier, Gianluca Severi, Marie Zins, Xavier de Lamballerie

**Affiliations:** 1 Institut Pierre-Louis d’Épidémiologie et de Santé Publique, Sorbonne Université, Inserm, Département de santé publique, Hôpital Saint-Antoine, APHP, 27 rue Chaligny, 75571 Paris Cedex 12, France; 2 Unité des Virus Émergents, UVE, IRD 190, INSERM 1207, Aix Marseille Univ, IHU Méditerranée Infection, Marseille, France; 3 Fondation Jean Dausset-CEPH (Centre d’Etude du Polymorphisme Humain), CEPH-Biobank, Paris, France; 4 Institut Pierre-Louis d’Épidémiologie et de Santé Publique, Sorbonne Université, Inserm, Paris, France; 5 Inserm U1153, Inrae U1125, Cnam, Nutritional Epidemiology Research Team (EREN), Sorbonne Paris Nord University, Epidemiology and Statistics Research Center – University of Paris (CRESS), Bobigny, France; 6 CESP UMR1018, UVSQ, Inserm, Université Paris-Saclay, Gustave Roussy, Villejuif, France; 7 Department of Statistics, Computer Science and Applications, University of Florence, Florence, Italy; 8 Paris University, Paris, France; 9 UVSQ, Inserm UMS 11, Université Paris-Saclay, Université de Paris, Villejuif, France

Correction to: *Scientific Reports* 10.1038/s41598-022-11787-4, published online 21 May 2022

Mathilde Touvier, Gianluca Severi, Marie Zins, Xavier de Lamballerie were omitted from the main author list in the original version of this Article.

The original Article has been corrected.

